# An updated HACOR score for predicting the failure of noninvasive ventilation: a multicenter prospective observational study

**DOI:** 10.1186/s13054-022-04060-7

**Published:** 2022-07-03

**Authors:** Jun Duan, Lijuan Chen, Xiaoyi Liu, Suha Bozbay, Yuliang Liu, Ke Wang, Antonio M. Esquinas, Weiwei Shu, Fuxun Yang, Dehua He, Qimin Chen, Bilin Wei, Baixu Chen, Liucun Li, Manyun Tang, Guodan Yuan, Fei Ding, Tao Huang, Zhongxing Zhang, ZhiJun Tang, Xiaoli Han, Lei Jiang, Linfu Bai, Wenhui Hu, Rui Zhang, Bushra Mina

**Affiliations:** 1 Department of Respiratory and Critical Care Medicine, The First Affiliated Hospital of Chongqing Medical University, Youyi Road 1, Yuzhong District, Chongqing, 400016 China; 2 Department of Respiratory and Critical Care Medicine, Sichuan Provincial People’s Hospital, University of Electronic Science and Technology of China, Chengdu, China; 3 Department of Critical Care Medicine, Dazhou Central Hospital, Dazhou, Shichuan China; 4 Intensive Care, Istanbul University Cerrahpasa-Cerrahpasa School of Medicine, Istanbul, Turkey; 5 Department of Respiratory and Critical Care Medicine, The Second Affiliated Hospital of Chongqing Medical University, Chongqing, China; 6 Intensive Care Unit, Hospital Morales Meseguer, Murcia, Spain; 7 Department of Critical Care Medicine, Yongchuan Hospital of Chongqing Medical University, Yongchuan, Chongqing, China; 8 Department of ICU, Sichuan Provincial People’s Hospital, University of Electronic Science and Technology of China, Chengdu, China; 9 Department of Critical Care Medicine, The Affiliated Hospital of Guizhou Medical University, Guiyang, Guizhou China; 10 Department of Critical Care Medicine, The First Affiliated Hospital, Sun Yat-Sen University, Guangzhou, China; 11 Department of Critical Care Medicine, West China Hospital of Sichuan University, Chengdu, Sichuan China; 12 Department of Respiratory and Critical Care Medicine, The Second Xiangya Hospital, Central South University, Changsha, Hunan China; 13 Department of Traditional Medicine and Rehabilitation, The Chest Hospital of Xi’an, Xi’an, China; 14 Department of Critical Care Medicine, Chonqing Public Health Medical Center, Chongqing, China; 15 Department of Respiratory and Critical Care Medicine, Bishan Hospital of Chongqing Medical University, Chongqing, China; 16 Department of Critical Care Medicine, The First Affiliated Hospital of Chongqing Medical University, Chongqing, China; 17 Department of Respiratory and Critical Care Medicine, Affiliated Hospital of Chongqing Three Gorges Medical College, Chongqing, China; 18 Department of Respiratory and Critical Care Medicine, The People’s Hospital of Nanchuan, Chongqing, China; 19 Department of Medicine, Division of Pulmonary and Critical Care Medicine, Northwell Health, Lenox Hill Hospital, New York, NY USA

**Keywords:** Noninvasive ventilation, Acute respiratory failure, Scoring system

## Abstract

**Background:**

Heart rate, acidosis, consciousness, oxygenation, and respiratory rate (HACOR) have been used to predict noninvasive ventilation (NIV) failure. However, the HACOR score fails to consider baseline data. Here, we aimed to update the HACOR score to take into account baseline data and test its predictive power for NIV failure primarily after 1–2 h of NIV.

**Methods:**

A multicenter prospective observational study was performed in 18 hospitals in China and Turkey. Patients who received NIV because of hypoxemic respiratory failure were enrolled. In Chongqing, China, 1451 patients were enrolled in the training cohort. Outside of Chongqing, another 728 patients were enrolled in the external validation cohort.

**Results:**

Before NIV, the presence of pneumonia, cardiogenic pulmonary edema, pulmonary ARDS, immunosuppression, or septic shock and the SOFA score were strongly associated with NIV failure. These six variables as baseline data were added to the original HACOR score. The AUCs for predicting NIV failure were 0.85 (95% CI 0.84–0.87) and 0.78 (0.75–0.81) tested with the updated HACOR score assessed after 1–2 h of NIV in the training and validation cohorts, respectively. A higher AUC was observed when it was tested with the updated HACOR score compared to the original HACOR score in the training cohort (0.85 vs. 0.80, 0.86 vs. 0.81, and 0.85 vs. 0.82 after 1–2, 12, and 24 h of NIV, respectively; all *p* values < 0.01). Similar results were found in the validation cohort (0.78 vs. 0.71, 0.79 vs. 0.74, and 0.81 vs. 0.76, respectively; all *p* values < 0.01). When 7, 10.5, and 14 points of the updated HACOR score were used as cutoff values, the probability of NIV failure was 25%, 50%, and 75%, respectively. Among patients with updated HACOR scores of ≤ 7, 7.5–10.5, 11–14, and > 14 after 1–2 h of NIV, the rate of NIV failure was 12.4%, 38.2%, 67.1%, and 83.7%, respectively.

**Conclusions:**

The updated HACOR score has high predictive power for NIV failure in patients with hypoxemic respiratory failure. It can be used to help in decision-making when NIV is used.

**Supplementary Information:**

The online version contains supplementary material available at 10.1186/s13054-022-04060-7.

## Introduction

Noninvasive ventilation (NIV) reduces inspiratory muscle effort and improves oxygenation in hypoxemic patients with acute respiratory failure [1]. As it offers several major advantages over invasive ventilation (e.g., preserving the ability to swallow, cough, and communicate verbally), NIV is widely used to avoid intubation [2]. However, the rate of NIV failure is 40–54% in hypoxemic patients [3–6]. Moreover, NIV failure is associated with increased mortality [7, 8]. Among patients who experience NIV failure, late failure further increases mortality [5, 9]. Therefore, early identification of patients at high risk for NIV failure and early application of invasive ventilation may reduce mortality.

Our team previously developed a scale that produces the HACOR score, which takes into account heart rate, acidosis, consciousness, oxygenation, and respiratory rate (Additional file 1: Table 1), to predict NIV failure in patients with hypoxemic respiratory failure [5]. This scale was developed based on data from a respiratory intensive care unit (ICU). Although it has high predictive power for NIV failure, extensive use of the scale may be limited by the fact that the majority of respiratory failure results from respiratory etiology. Furthermore, baseline data such as the presence of acute respiratory distress syndrome (ARDS), septic shock, immunosuppression, organ failure, and so on are also associated with NIV failure [6, 7, 10, 11]. Because these baseline data may improve the predictive power of the score, we aimed to incorporate them into the HACOR score to improve its predictive power for NIV failure in patients with hypoxemic respiratory failure.

## Methods

This multicenter prospective observational study was performed in 17 hospitals in China from September 2017 to September 2021 and one hospital in Turkey from November 2018 to August 2020. The study protocol was approved by the ethics committee of the First Affiliated Hospital of Chongqing Medical University (No. 2016150) and the ethics committee of Istanbul University Cerrahpasa (No. 88295). Informed consent was obtained from patients or their family members.

Patients admitted to the ICU for NIV due to hypoxemic respiratory failure were enrolled. However, patients who were younger than 16 years old, who experienced hypercapnic respiratory failure, who required emergency intubation, who underwent the use of NIV after extubation, who received NIV after accidental extubation, and who received NIV because of acute exacerbation of chronic obstructive pulmonary disease were excluded. Patients who received NIV because of high-flow nasal cannula failure or had undergone NIV more than 2 h before being admitted to the participating center were also excluded. Emergency intubation means that intubation was required immediately because the patient was in respiratory or cardiac arrest, was experiencing respiratory pauses with loss of consciousness, or was gasping for air.

All patients who used NIV were managed by attending physicians, respiratory therapists, and nurses in charge based on current guidelines, consensus, and previously published methods [5, 12–15]. The indications for NIV were as follows: (1) respiratory rate > 25 breaths/min, (2) clinical presentation of respiratory distress at rest (such as active contraction of the accessory inspiratory muscles or paradoxical abdominal motion), or (3) PaO_2_ < 60 mmHg at room air or PaO_2_/FiO_2_ < 300 mmHg with supplemental oxygen. If supplemental oxygen was used, FiO_2_ was estimated as follows: FiO_2_ (%) = 21 + 4 × flow (L/min) [16, 17]. However, the use of NIV was at the physician’s discretion. Continuous positive airway pressure (CPAP) or bilevel positive pressure ventilation was used to relieve patients’ dyspnea. Parameters were increased gradually based on patients’ tolerance. CPAP, expiratory positive airway pressure, or positive end expiratory pressure was usually maintained between 4 and 10 cmH_2_O. Inspiratory pressure was maintained between 10 and 20 cmH_2_O. The fractional concentration of oxygen was set to achieve peripheral oxygen saturation greater than 92%. In addition, appropriate strategies were used to improve NIV tolerance, such as controlling leakage, keeping the anchoring system as comfortable as possible, providing adequate humidification, alternatively using different interfaces, and administering sedation [18].

We encouraged patients to use NIV as long as possible initially. If their respiratory distress was relieved and oxygenation improved, NIV was used intermittently until patients could be completely liberated. If respiratory failure progressively deteriorated, intubation for invasive mechanical ventilation was performed. The major criteria for intubation were as follows: respiratory or cardiac arrest, PaO_2_/FiO_2_ < 100 mmHg after NIV intervention, the development of conditions necessitating intubation to protect the airway (coma or seizure disorders) or to manage copious tracheal secretions, and hemodynamic instability without response to fluids or vasoactive agents [5, 19]. Minor criteria were as follows: PaO_2_/FiO_2_ < 150 mmHg after NIV intervention, respiratory rate > 35 breaths/min, lack of improvement in respiratory muscle fatigue, and acidosis with pH < 7.35. Intubation was recommended if one major criterion or more than two minor criteria were reached. However, the decision to intubate was at the discretion of the attending physician. The need for intubation was defined as NIV failure [6].

We collected baseline data, vital signs, and arterial blood gas (ABG) from initiation to 24 h of NIV. Baseline data included ICU type, age, sex, reason for NIV, underlying disease, severity of disease (assessed by sequential organ failure assessment [SOFA] score), presence of COVID-19, presence of septic shock, and presence of ARDS. The presence of COVID-19 means that hypoxemic acute respiratory failure resulted from SARS-CoV-2 infection. Vital signs included consciousness (assessed by the Glasgow Coma Scale), heart rate, respiratory rate, systolic blood pressure, and diastolic blood pressure. The SOFA score was calculated before NIV. Urine output was obtained from medical records. If urine output was not available from medical records, it was estimated by the patient. Pneumonia was diagnosed based on current guidelines (i.e., a radiographic infiltrate that is new or progressive along with clinical findings suggesting infection, including the new onset of fever, purulent sputum, leukocytosis, shortness of breath, and a decline in oxygenation) [20, 21].

The aim of the current study was to update the HACOR score to predict NIV failure in hypoxemic patients. We used data collected in nine hospitals in Chongqing, China (*N* = 1451), to train the scale (training cohort). Data from another eight hospitals elsewhere in China and one hospital in Turkey (*N* = 728) were used to validate the scale (external validation cohort). The current reporting is based on transparent reporting of a multivariable prediction model for individual prognosis or diagnosis [22].

### Statistical analysis

We used SPSS (version 25.0) and R (version 4.0.5) to analyze the data. Given an estimated NIV failure rate of 44% and estimated sensitivity and specificity of more than 70% (assuming the expected standard error of 5%), at least 734 patients were required to update the HACOR score for *α* = 0.05 [5]. Multiple imputations were performed to address missing data. The area under the receiver operating characteristic curve (AUC) was used to analyze the predictive power of NIV failure. A *p* value less than 0.05 was considered to be statistically significant.

The data from the training cohort were used to update the HACOR score. First, we selected variables via elastic net regularization, using logistic models and tenfold cross-validation, selecting the regularization parameter λ when binomial deviation was within one standard error of the minimum [23, 24]. Collinearity between continuous variables was identified if the absolute value of the correlation coefficients was > 0.7 [25]. The selected variables were used to develop a basic score for predicting NIV failure. Then, we combined this basic score and the original HACOR score to create the updated HACOR score. The final model for goodness of fit was tested using the Hosmer–Lemeshow test. Details of the development of the updated HACOR score in the training cohort can be seen in Additional file 1: Method 1. The predictive powers for NIV failure of the original and updated HACOR scores were compared with the Hanley and McNeil method [26]. For clinical reference, three cutoff values were selected for probabilities of NIV failure equal to 25%, 50%, and 75% [27]. Probabilities of NIV failure of less than 25%, 25–50%, 50–75%, and more than 75% were defined as low, moderate, high, and very high risk for NIV failure, respectively. According to the original HACOR study, patients with HACOR scores ≤ 5 and > 5 were defined as being at low and high risk for NIV failure, respectively [5].

## Results

### Demographic characteristics

The flow of patient screening is summarized in Additional file 1: Fig. 1. In the training cohort, 24 patients had missing data (0.2% for ABG before NIV, 1.4% for ABG after 1–2 h of NIV, and 0.07% for SOFA score). In the validation cohort, 18 patients had missing data (0.1% for ABG before NIV, 0.1% for heart rate before NIV, and 2.2% for ABG after 1–2 h of NIV). All missing data were interpolated by multiple imputations.

In the training cohort, 529 patients (36.5%) experienced NIV failure (Table 1). In the validation cohort, 328 patients (45.1%) experienced NIV failure. In both cohorts, about half of patients were from medical ICUs, one-third were from mixed ICUs, and the rest were from surgical ICUs.

**Table 1:** Demographic characteristics

	Training cohort		*p*^a^	Validation cohort		*p*^a^	*p*^b^
	NIV success *N* = 922	NIV failure *N* = 529		NIV success *N* = 400	NIV failure *N* = 328		
Age, years	63 ± 16	64 ± 16	0.61	61 ± 17	61 ± 16	0.99	< 0.01
Male	593 (64.3%)	377 (71.3%)	< 0.01	273 (68.3%)	217 (66.2%)	0.58	0.85
SOFA score	4.6 ± 2.4	6.1 ± 3.0	< 0.01	4.9 ± 2.1	6.0 ± 2.8	< 0.01	0.03
Septic shock	67 (7.3%)	138 (26.1%)	< 0.01	14 (3.5%)	59 (18.0%)	< 0.01	< 0.01
Presence of COVID-19	4 (0.4%)	0 (0%)	0.30	17 (4.3%)	26 (7.9%)	0.04	< 0.01
ICU type							
Medical	450 (48.8%)	300 (56.7%)	< 0.01	179 (44.8%)	151 (46.0%)	0.77	0.19
Surgical	145 (15.7%)	58 (11.0%)	0.01	58 (14.5%)	50 (15.2%)	0.83	0.60
Mixed	327 (35.5%)	171 (32.3%)	0.23	163 (40.8%)	127 (38.7%)	0.60	0.01
Reason for NIV							
Pneumonia	363 (39.4%)	347 (65.6%)	< 0.01	230 (57.5%)	255 (77.7%)	< 0.01	< 0.01
Nonpulmonary sepsis	113 (12.3%)	79 (14.9%)	0.15	29 (7.3%)	27 (8.2%)	0.68	< 0.01
Pancreatitis	109 (11.8%)	32 (6.0%)	< 0.01	23 (5.8%)	8 (2.4%)	0.04	< 0.01
CPE	131 (14.2%)	7 (1.3%)	< 0.01	40 (10.0%)	4 (1.2%)	< 0.01	< 0.01
Cardiac problem other than CPE	58 (6.3%)	16 (3.0%)	< 0.01	11 (2.8%)	10 (3.0%)	0.83	0.02
Pulmonary embolism	16 (1.7%)	10 (1.9%)	0.84	8 (2.0%)	3 (0.9%)	0.36	0.73
Trauma	19 (2.1%)	5 (0.9%)	0.14	13 (3.3%)	5 (1.5%)	0.16	0.19
Poison	8 (0.9%)	5 (0.9%)	> 0.99	3 (0.8%)	0 (0%)	0.26	0.29
Postoperative respiratory failure	9 (1.0%)	3 (0.6%)	0.55	9 (2.3%)	3 (0.9%)	0.24	0.13
Asthma	7 (0.8%)	0 (0%)	0.05	1 (0.3%)	0 (0%)	> 0.99	0.28
Inhalation injury	4 (0.4%)	0 (0%)	0.30	1 (0.3%)	1 (0.3%)	> 0.99	> 0.99
Other	85 (9.2%)	25 (4.7%)	< 0.01	32 (8.0%)	12 (3.7%)	0.02	0.22
Presence of ARDS							
Pulmonary ARDS	49 (5.3%)	102 (19.3%)	< 0.01	48 (12.0%)	78 (23.8%)	< 0.01	< 0.01
Extrapulmonary ARDS	109 (11.8%)	51 (9.6%)	0.22	9 (2.3%)	8 (2.4%)	> 0.99	< 0.01
Underlying disease							
Hypertension	394 (42.7%)	193 (36.5%)	0.02	166 (41.5%)	102 (31.3%)	< 0.01	0.10
Diabetes mellitus	261 (28.3%)	130 (24.6%)	0.13	91 (22.8%)	68 (20.7%)	0.53	0.01
Chronic kidney disease	109 (11.8%)	41 (7.8%)	0.02	73 (18.3%)	41 (14.5%)	0.04	< 0.01
Chronic liver disease	24 (2.6%)	22 (4.2%)	0.12	21 (5.3%)	23 (7.0%)	0.35	< 0.01
Chronic heart disease	300 (32.5%)	109 (20.6%)	< 0.01	119 (29.8%)	84 (25.6%)	0.25	0.92
Chronic lung disease	143 (15.5%)	69 (13.0%)	0.22	60 (15.0%)	40 (12.2%)	0.28	0.60
Presence of immunosuppression	45 (4.9%)	72 (13.6%)	< 0.01	60 (15.0%)	81 (24.7%)	< 0.01	< 0.01
Variables collected before NIV							
GCS	14.8 ± 0.6	14.6 ± 1.0	< 0.01	14.4 ± 1.5	14.2 ± 2.2	0.07	< 0.01
Heart rate, beats/min	114 ± 24	118 ± 24	< 0.01	106 ± 23	111 ± 23	< 0.01	< 0.01
Respiratory rate, breaths/min	30 ± 7	33 ± 8	< 0.01	28 ± 6	30 ± 8	< 0.01	< 0.01
Systolic blood pressure, mmHg	134 ± 27	129 ± 26	< 0.01	134 ± 24	128 ± 25	< 0.01	0.49
Diastolic blood pressure, mmHg	78 ± 17	75 ± 17	0.02	78 ± 17	74 ± 15	< 0.01	0.52
pH	7.42 ± 0.10	7.41 ± 0.12	0.11	7.43 ± 0.08	7.42 ± 0.10	0.04	0.11
PaCO_2_, mmHg	33 ± 8	32 ± 8	0.01	34 ± 7	34 ± 8	0.67	< 0.01
PaO_2_/FiO_2_, mmHg	178 ± 74	153 ± 60	< 0.01	147 ± 104	137 ± 85	0.16	< 0.01
HACOR score	4.7 ± 2.7	6.4 ± 3.1	< 0.01	5.7 ± 3.1	6.9 ± 3.5	< 0.01	< 0.01
Variables collected after 1–2 h of NIV							
GCS	14.9 ± 0.4	14.5 ± 1.2	< 0.01	14.5 ± 1.5	14.2 ± 2.2	0.03	< 0.01
Heart rate, beats/min	105 ± 22	112 ± 24	< 0.01	100 ± 21	107 ± 22	< 0.01	< 0.01
Respiratory rate, breaths/min	26 ± 6	31 ± 8	< 0.01	25 ± 6	28 ± 8	< 0.01	< 0.01
Systolic blood pressure, mmHg	129 ± 23	127 ± 25	0.19	128 ± 22	124 ± 22	0.01	0.05
Diastolic blood pressure, mmHg	73 ± 14	71 ± 16	0.06	76 ± 15	73 ± 14	0.01	< 0.01
pH	7.44 ± 0.07	7.40 ± 0.11	< 0.01	7.44 ± 0.06	7.42 ± 0.09	0.01	0.22
PaCO_2_, mmHg	34 ± 7	34 ± 12	0.38	35 ± 7	35 ± 9	0.70	0.03
PaO_2_/FiO_2_, mmHg	241 ± 108	168 ± 89	< 0.01	182 ± 67	140 ± 61	< 0.01	< 0.01
HACOR score	2.5 ± 2.2	6.0 ± 3.6	< 0.01	3.9 ± 2.9	6.2 ± 3.5	< 0.01	< 0.01
De novo acute respiratory failure	508 (55.1%)	358 (69.6%)	< 0.01	233 (58.3%)	215 (65.5%)	< 0.05	0.93
Outcome							
Death in hospital	53 (5.7%)	256 (48.4%)	< 0.01	15 (3.8%)	153 (46.6%)	< 0.01	0.35
Length of ICU stay, days	6 (4–10)	7 (3–13)	0.14	7 (5–12)	9 (4–16)	0.20	< 0.01
Length of hospital stay, days	16 (10–26)	11 (5–22)	< 0.01	18 (11–28)	14 (6–24)	< 0.01	0.02

*NIV*  noninvasive ventilation, *SOFA *  sequential organ failure assessment,* ICU*  intensive care unit, *CPE *  cardiogenic pulmonary edema, *ARDS*  acute respiratory distress syndrome, *GCS* Glasgow Coma Scale,* HACOR* heart rate, acidosis, consciousness, oxygenation, and respiratory rate

^a^*p* for the difference between NIV success versus failure

^b^*p* for the difference between training cohort versus validation cohort

In the training cohort, patients who experienced NIV failure were more likely to have septic shock, pneumonia, pulmonary ARDS, hypertension, chronic kidney disease, and immunosuppression compared to those who experienced successful NIV. However, they were less likely to have pancreatitis and cardiogenic pulmonary edema (CPE). These results were confirmed in the validation cohort.

### Development of the updated HACOR score in the training cohort

Details of the development of the updated HACOR score are summarized in Additional file 1: Method 1. A diagnosis of pneumonia was a risk factor for NIV failure, and a diagnosis of CPE was a protective factor identified by elastic net logistic regression (Additional file 1: Figs. 2 and 3). The presence of pulmonary ARDS, immunosuppression, or septic shock and the SOFA score before NIV were risk factors for NIV failure. Thus, we updated the HACOR score to take these six pre-NIV variables into account (Additional file 1: Tables 2–4).

Therefore, the updated HACOR score is as follows: original HACOR score + 0.5 × SOFA + 2.5 if pneumonia is diagnosed – 4 if CPE is diagnosed + 3 if pulmonary ARDS is present + 1.5 if immunosuppression is present + 2.5 if septic shock is present. The *p* value for goodness of fit was 0.21 when the Hosmer–Lemeshow test was used. This indicates that the final model was properly fitted.

### The predictive powers for NIV failure of the original and updated HACOR scores

In both cohorts, the AUCs for predicting NIV failure were higher when tested by the updated HACOR score than the original HACOR score from initiation to 24 h of NIV (all *p* values < 0.01; Table 2). The AUCs for predicting NIV failure were 0.85 (95% confidence interval 0.84–0.87) and 0.78 (0.75–0.81) tested by the updated HACOR score assessed after 1–2 h of NIV in the training and validation cohorts, respectively. The AUCs in the different subgroups are summarized in Table 3.

**Table 2:** AUCs for the HACOR and updated HACOR scores for predicting NIV failure

	Training cohort	*p*^#^	Validation cohort	*p*^#^
Before NIV	*N* = 1451		*N* = 728	
HACOR	0.67 (0.64–0.69)	< 0.01	0.62 (0.58–0.65)	< 0.01
Updated HACOR	0.78 (0.76–0.80)		0.71 (0.67–0.74)	
After 1–2 h of NIV	*N* = 1451		*N* = 728	
HACOR	0.80 (0.78–0.82)	< 0.01	0.71 (0.68–0.74)	< 0.01
Updated HACOR	0.85 (0.84–0.87)		0.78 (0.75–0.81)	
After 12 h of NIV	*N* = 1133		*N* = 633	
HACOR	0.81 (0.79–0.84)	< 0.01	0.74 (0.70–0.77)	< 0.01
Updated HACOR	0.86 (0.83–0.88)		0.79 (0.76–0.82)	
After 24 h of NIV	*N* = 942		*N* = 552	
HACOR	0.82 (0.79–0.84)	< 0.01	0.76 (0.72–0.80)	< 0.01
Updated HACOR	0.85 (0.83–0.88)		0.81 (0.77–0.84)	

*AUC*  area under the receiver operating characteristic curve, *HACOR*  heart rate, acidosis, consciousness, oxygenation, and respiratory rate,* NIV*  noninvasive ventilation

^#^*p* for the difference in AUC between HACOR versus updated HACOR

**Table 3:** AUCs for the updated HACOR score for predicting NIV failure in different subgroups

	After 1–2 h of NIV AUC (95% CI)	After 12 h of NIV AUC (95% CI)	After 24 h of NIV AUC (95% CI)
Training cohort			
De novo acute respiratory failure, *N* = 876^#^	0.83 (0.81–0.86)	0.84 (0.81–0.87)	0.85 (0.81–0.88)
Pneumonia, *N* = 710	0.82 (0.79–0.85)	0.84 (0.81–0.88)	0.83 (0.80–0.87)
Pulmonary ARDS, *N* = 151	0.81 (0.74–0.88)	0.83 (0.76–0.90)	0.89 (0.82–0.95)
Extrapulmonary ARDS, *N* = 160	0.86 (0.80–0.93)	0.85 (0.77–0.93)	0.86 (0.78–0.95)
CPE, *N* = 138	0.81 (0.63–1.00)	0.92 (0.80–1.00)	0.80 (0.53–1.00)
Cardiac problem other than CPE, *N* = 74	0.76 (0.60–0.92)	0.78 (0.63–0.92)	0.84 (0.69–0.99)
Nonpulmonary sepsis, *N* = 192	0.83 (0.78–0.89)	0.76 (0.66–0.85)	0.71 (0.59–0.83)
Pancreatitis, *N* = 141	0.81 (0.72–0.91)	0.84 (0.75–0.93)	0.88 (0.79–0.96)
Validation cohort			
De novo acute respiratory failure, *N* = 438^#^	0.76 (0.71–0.80)	0.77 (0.73–0.82)	0.80 (0.75–0.85)
Pneumonia, *N* = 485	0.73 (0.69–0.77)	0.75 (0.71–0.80)	0.77 (0.72–0.81)
Pulmonary ARDS, *N* = 126	0.75 (0.66–0.83)	0.74 (0.65–0.84)	0.74 (0.64–0.84)
Extrapulmonary ARDS, *N* = 17	0.74 (0.49–0.99)	0.92 (0.75–1.00)	1.00 (1.00–1.00)
CPE, *N* = 44	0.78 (0.53–1.00)	0.88 (0.75–1.00)	0.89 (0.74–1.00)
Cardiac problem other than CPE, *N* = 21	0.95 (0.86–1.00)	0.80 (0.57–1.00)	1.00 (1.00–1.00)
Nonpulmonary sepsis, *N* = 56	0.83 (0.71–0.94)	0.75 (0.60–0.90)	0.75 (0.58–0.92)
Pancreatitis, *N* = 31	0.89 (0.78–1.00)	0.71 (0.48–0.94)	0.97 (0.91–1.00)
COVID-19, *N* = 43	0.78 (0.63–0.92)	0.70 (0.54–0.86)	0.82 (0.68–0.96)

*AUC*  area under the receiver operating characteristic curve, *HACOR *  heart rate, acidosis, consciousness, oxygenation, and respiratory rate, *NIV*  noninvasive ventilation, *CI*  confidence interval, *ARDS*  acute respiratory distress syndrome,* CPE*  cardiogenic pulmonary edema

^#^De novo acute respiratory failure is defined as the occurrence of respiratory failure without chronic respiratory disease, chronic heart disease, asthma, CPE, cardiac problem other than CPE, or postoperative hypoxemia

**Fig. 1:**
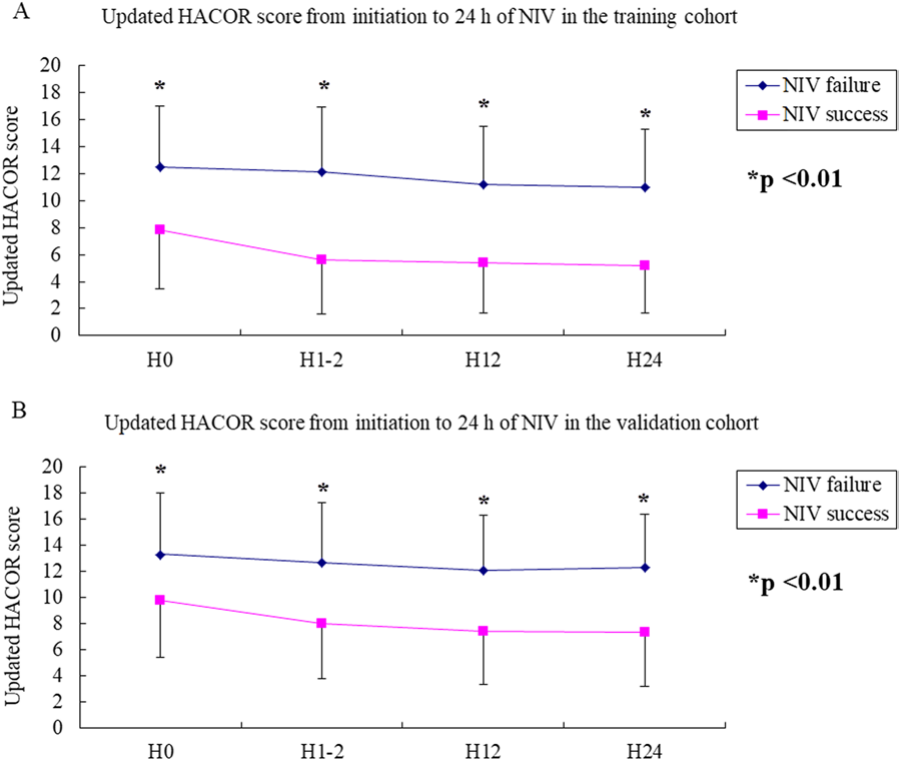
Updated HACOR scores of patients with successful NIV and NIV failure from initiation to 24 h of NIV. Data are means and standard deviations. *p < 0.01 for the comparison of patients with successful NIV versus NIV failure. H0 = before NIV, H1-2 = after 1–2 h of NIV, H12 = after 12 h of NIV, H24 = after 24 h of NIV, NIV = noninvasive ventilation, HACOR = heart rate, acidosis, consciousness, oxygenation, and respiratory rate

**Fig. 2:**
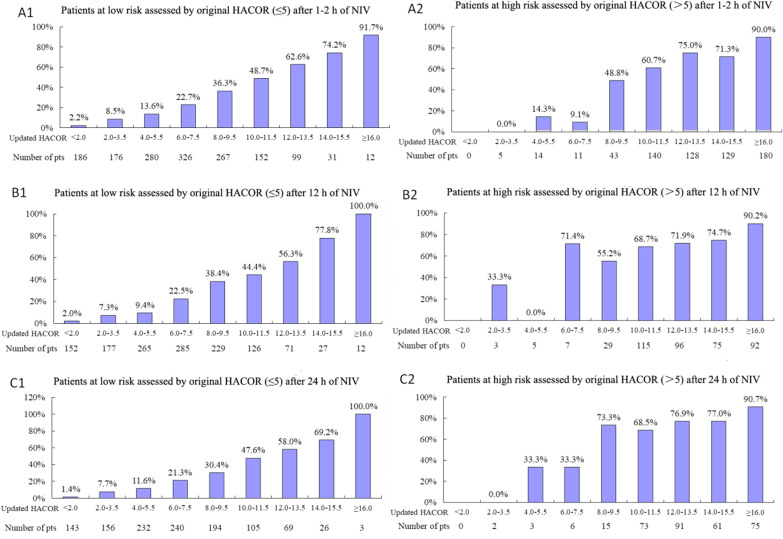
Rate of NIV failure within 24 h of NIV. A1, B1, and C1 indicate the rate of NIV failure in different subgroups classified by updated HACOR scores among patients with an original HACOR score ≤ 5. A2, B2, and C2 indicate the rate of NIV failure in different subgroups classified by updated HACOR scores among patients with an original HACOR score > 5. NIV = noninvasive ventilation, HACOR = heart rate, acidosis, consciousness, oxygenation, and respiratory rate

From initiation to 24 h of NIV, the updated HACOR score was much higher in patients who experienced NIV failure than those who experienced successful NIV (Fig. 1). The rate of NIV failure increased with an increase in the updated HACOR score, whether it was assessed before NIV or after 1–2, 12, or 24 h of NIV (Additional file 1: Figs. 4 and 5). In patients at low risk as assessed by the original HACOR score, the rate of NIV failure was greater than 50% if the updated HACOR score was more than 12 (Fig. 2). In contrast, in patients at high risk as assessed by the original HACOR score, the rate of NIV failure was low in most cases if the updated HACOR score was less than 8.

**Fig. 3:**
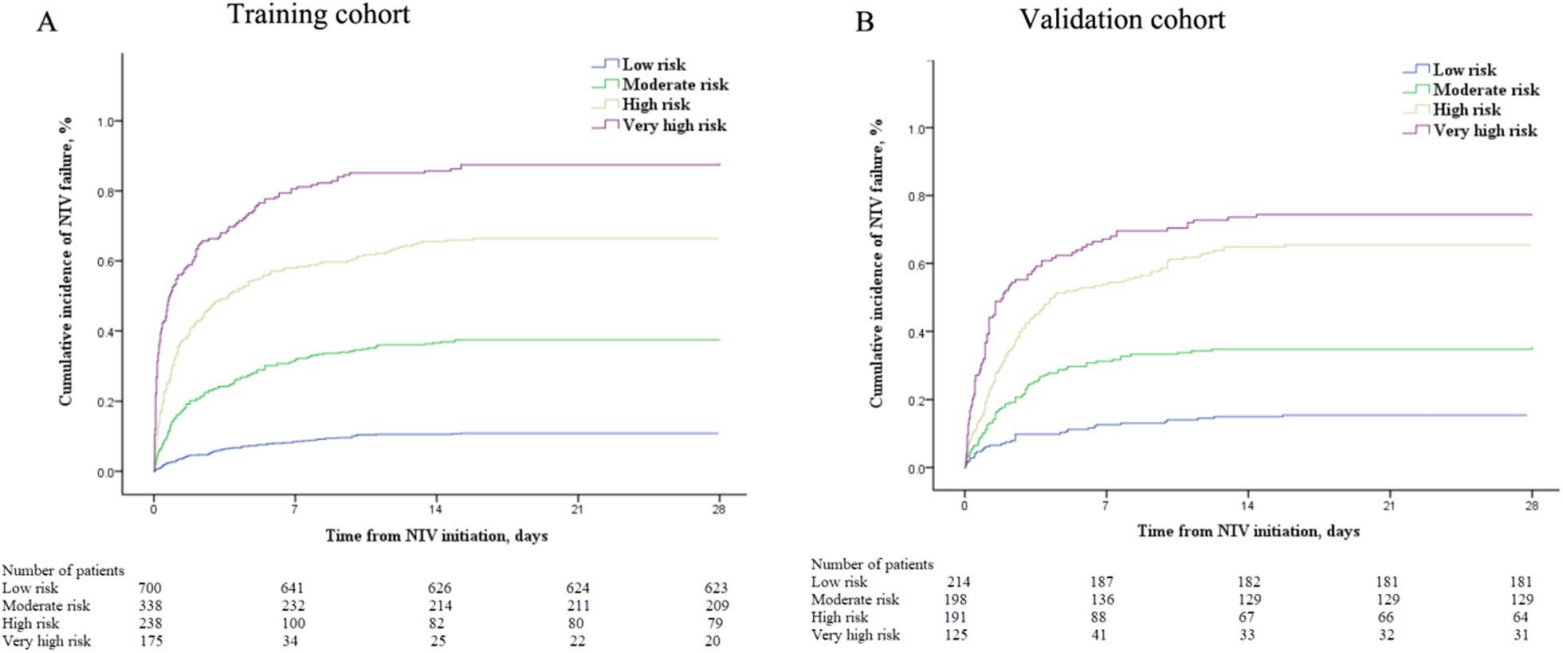
Cumulative incidence of NIV failure in patients at low, moderate, high, and very high risk for NIV failure when the updated HACOR score is assessed after 1–2 h of NIV. Patients with updated HACOR scores of ≤ 7, 7.5–10.5, 11–14, and > 14, respectively, were classified as being at low, moderate, high, and very high risk for NIV failure. NIV = noninvasive ventilation, HACOR = heart rate, acidosis, consciousness, oxygenation, and respiratory rate

When 7, 10.5, and 14 points of updated HACOR score were selected as cutoff values, the probability of NIV failure was 25%, 50%, and 75%, respectively. The predictive power is reported in Table 4. Using the three cutoff values, we classified patients as being at low (≤ 7), moderate (7.5–10.5), high (11–14), and very high (> 14) risk for NIV failure. The cumulative incidence of NIV failure is summarized in Fig. 3. For all patients, the rate of NIV failure was 12.4%, 38.2%, 67.1%, and 83.7% among patients with a low, moderate, high, and very high probability of NIV failure, respectively.

**Table 4:** Predictive power for NIV failure of the updated HACOR score

Cutoff value	SE	SP	PPV	NPV	+ LR	−LR
*Training cohort*						
After 1–2 h of NIV, *N* = 1451						
> 7	84.9%	67.3%	59.8%	88.6%	2.59	0.22
> 10.5	59.9%	89.6%	76.8%	79.6%	5.76	0.45
> 14	29.5%	97.9%	89.1%	70.8%	14.31	0.72
After 12 h of NIV, *N* = 1133						
> 7	84.0%	71.2%	58.5%	90.2%	2.92	0.22
> 10.5	55.0%	91.8%	76.3%	80.9%	6.67	0.49
> 14	20.9%	99.5%	95.1%	72.2%	39.86	0.80
After 24 h of NIV, *N* = 942						
> 7	77.9%	73.5%	56.6%	88.2%	2.94	0.30
> 10.5	51.7%	93.4%	77.7%	81.3%	7.84	0.52
> 14	21.0%	99.2%	92.4%	73.9%	27.4	0.80
*Validation cohort*						
After 1–2 h of NIV, *N* = 728						
> 7	89.9%	45.3%	57.4%	84.6%	1.64	0.22
> 10.5	67.7%	76.5%	70.3%	74.3%	2.88	0.42
> 14	29.0%	92.5%	76.0%	61.4%	3.86	0.77
After 12 h of NIV, *N* = 633						
> 7	90.5%	51.2%	56.7%	88.4%	1.85	0.19
> 10.5	60.3%	79.0%	66.9%	73.8%	2.87	0.50
> 14	27.5%	95.2%	80.0%	65.0%	5.66	0.76
After 24 h of NIV, *N* = 552						
> 7	90.5%	53.2%	56.3%	89.3%	1.93	0.18
> 10.5	66.1%	78.3%	67.0%	77.5%	3.04	0.43
> 14	23.5%	95.2%	76.5%	65.1%	4.87	0.80

*NIV*  noninvasive ventilation, *HACOR*  heart rate, acidosis, consciousness, oxygenation, and respiratory rate, *SE*  sensitivity, *SP*  specificity, *PPV *  positive predictive value, *NPV *  negative predictive value, + *LR *  positive likelihood ratio, –*LR* negative likelihood ratio

## Discussion

The current study evaluates and confirms the power of an updated HACOR score that incorporates data on six baseline variables to predict NIV failure. The predictive power for NIV failure tested by the updated HACOR score was significantly improved compared to that of the original HACOR score. Three cutoff values indicating low, moderate, high, and very high probability of NIV failure were developed to aid clinical staff in decision-making.

The original HACOR score assessed heart rate, acidosis, consciousness, oxygenation, and respiratory rate [5]. The predictive power for NIV failure was high in the original study. However, the predictive power in the current study was not as good. In patients at low risk as assessed by the original HACOR score, the rate of NIV failure was high if the updated HACOR score was high. In contrast, in patients at high risk as assessed by the original HACOR score, the rate of NIV failure was low in most cases if the updated HACOR score was low. The reasons for this are as follows: The original HACOR score was developed based on vital signs and ABG results only. However, patients with different baseline data have different risks for NIV failure even when they have similar vital signs and ABG results. The presence of pulmonary ARDS, septic shock, immunosuppression, and organ failure at baseline are associated with NIV failure [6, 7, 10, 11, 28]. Moreover, patients with CPE have a very low rate of NIV failure, which acts as a protective factor against NIV failure [29, 30]. In this study, we incorporated these pre-NIV variables into the original HACOR score to update the score. That is why the predictive power was significantly improved.

In addition, the original HACOR score was developed and validated using data from a respiratory ICU. All patients had a respiratory etiology and were managed by respiratory physicians. However, the updated HACOR score was developed in nine hospitals and validated in another nine hospitals. The patients had different etiologies, came from different ICUs, and were managed by different physicians. Therefore, the patients and physicians in the updated HACOR study were more representative of the real world. This is another reason for the better predictive power of the updated HACOR score than the original one.

The use of NIV in patients with de novo acute respiratory failure, pneumonia, or ARDS is controversial because of the high risk for NIV failure [3, 4, 9, 30]. In some cases, the rate of NIV failure can reach 70% [31]. Guidelines contain no recommendations for using NIV with these patients [12]. In our study, the updated HACOR score had high predictive power for NIV failure in these patients whether it was assessed after 1–2, 12, or 24 h of NIV. A higher updated HACOR score indicates a higher risk for NIV failure. Therefore, the updated HACOR score provides an important reference point for clinical staff managing NIV. In patients at high risk for NIV failure identified by the updated HACOR score, early intubation can be considered.

A good risk scoring system can help clinical staff manage their patients. In our study, the updated HACOR score assessed after 1–2 h of NIV had high predictive power for NIV failure. It can identify patients who are more likely to experience intubation in the future. As late NIV failure is associated with increased mortality [5, 9], close monitoring, more staffing, and better device supply may benefit high-risk patients.

In routine clinical work, physicians may partly refer to the values of PaO_2_/FiO_2_ ratio, respiratory rate, and pH before they decided intubation. However, how to combine these variables and other risk factors together to make more suitable decision-making is difficult. One may consider more in PaO_2_/FiO_2_ ratio, and the other may consider more in respiratory rate. In other words, the weight is different for different physicians. The main contribution of the updated HACOR score is to calculate the weight in each variable. It took into account the major risk factors and quantitatively calculated the weight in each variable. Although the updated HACOR score may be explained by self-fulfilling prophecy in part, it provides more useful information for physicians to make reasonable decision.

This study has several limitations. First, we enrolled only 47 patients with COVID-19 in this study. This small subsample size may diminish the predictive power for NIV failure. Clinicians should be cautious when assessing the updated HACOR score in COVID-19 patients. Second, although we suggested intubation criteria, the decision to intubate was at the discretion of the attending physician. However, this reflects true conditions in the real world and thus may partly improve the generalizability of the updated HACOR score. Third, the benefit of an updated HACOR score is unclear, as the current study was observational in design. The effects of an updated HACOR score should be strictly demonstrated in randomized controlled trials. Fourth, we did not predefine the interface or a sedation plan. These issues were determined by the attending physicians in charge. Whether the interface or sedation plan is associated with NIV failure is unclear. Fifth, the expired tidal volume (standardization to predicted body weight) is associated with NIV failure [4]. In our study, most of the ventilators have used single-limb circuit. It is unable to measure expired tidal volume. In addition, the predicted body weight is unavailable because we did not record the patient’s height. So, we did not include the tidal volume in the model.

## Conclusions

The updated HACOR score, which combines data on six baseline variables and the five original scale items, has significantly improved predictive power for NIV failure compared to the original HACOR score. A higher score indicates a higher risk for NIV failure. Patients with updated HACOR scores of ≤ 7, 7.5–10.5, 11–14, and > 14, respectively, were classified as having a low, moderate, high, and very high probability of NIV failure. This updated score provides a reference for clinical staff in decision-making.

## Supplementary Information

**Additional file 1**. **Supplementary Table 1**. Points for each variable in the original HACOR score. **Supplementary Table 2**. Variables left in the elastic net logistic regression for predicting NIV failure in the training cohort from baseline data. **Supplementary Table 3**. Basic score for predicting NIV failure in the training cohort. **Supplementary Table 4**. Combining the basic score and the original HACOR score to predict NIV failure in the training cohort. **Supplementary Figure 1**. Flow of patient screening and enrollment. **Supplementary Figure 2**. Change in variable counts in the elastic net logistic regression. **Supplementary Figure 3**. The selection of variables in the elastic net logistic regression by 10-fold cross-validation. **Supplementary Figure 4**. Rate of NIV failure in patients in the training cohort with different updated HACOR scores. **Supplementary Figure 5**. Rate of NIV failure in patients in the validation cohort with different updated HACOR scores. **Supplementary Method 1**. Details of the development of the updated heart rate, acidosis, consciousness, oxygenation, and respiratory rate (HACOR) score in the training cohort.

## Data Availability

The dataset used and/or analyzed during the current study is available from the corresponding author on reasonable request.

## Abbreviations

HACOR: Heart rate, acidosis, consciousness, oxygenation, and respiratory rate
NIV: Noninvasive ventilation
AUC: Area under the receiver operating characteristic curve
CI: Confidence interval
OR: Odds ratio
ARDS: Acute respiratory distress syndrome
CPE: Cardiogenic pulmonary edema
SOFA: Sequential organ failure assessment
ICU: Intensive care unit
GCS: Glasgow Coma Scale
SE: Sensitivity
SP: Specificity
PPV: Positive predictive value
NPV: Negative predictive value
+ LR: Positive likelihood ratio
–LR: Negative likelihood ratio
