# Supplementary material for: An updated HACOR score for predicting the failure of noninvasive ventilation: a multicenter prospective observational study
Source: Crit Care. 2022 Jul 3;26:196. doi: 10.1186/s13054-022-04060-7 (PMC9250742; doi:10.1186/s13054-022-04060-7)
Supplement: Supplementary file 1 — Additional file 1. Supplementary Table 1. Points for each variable in the original HACOR score. Supplementary Table 2. Variables left in the elastic net logistic regression for predicting NIV failure in the training cohort from baseline data. Supplementary Table 3. Basic score for predicting NIV failure in the training cohort. Supplementary Table 4. Combining the basic score and the original HACOR score to predict NIV failure in the training cohort. Supplementary Figure 1. Flow of patient screening and enrollment. Supplementary Figure 2. Change in variable counts in the elastic net logistic regression. Supplementary Figure 3. The selection of variables in the elastic net logistic regression by 10-fold cross-validation. Supplementary Figure 4. Rate of NIV failure in patients in the training cohort with different updated HACOR scores. Supplementary Figure 5. Rate of NIV failure in patients in the validation cohort with different updated HACOR scores. Supplementary Method 1. Details of the development of the updated heart rate, acidosis, consciousness, oxygenation, and respiratory rate (HACOR) score in the training cohort. [file 13054_2022_4060_MOESM1_ESM.docx]

Supplementary Table 1. Points for each variable in the original HACOR score^#^

| Variable | Category | Points |
| --- | --- | --- |
| Heart rate, beats/min | <120 | 0 |
|  | ≥121 | 1 |
| pH (acidosis) | ≥7.35 | 0 |
|  | 7.30–7.34 | 2 |
|  | 7.25–7.29 | 3 |
|  | <7.25 | 4 |
| GCS (consciousness) | 15 | 0 |
|  | 13–14 | 2 |
|  | 11–12 | 5 |
|  | ≤10 | 10 |
| PaO_2_/FiO_2_ (oxygenation), mmHg | ≥201 | 0 |
|  | 176–200 | 2 |
|  | 151–175 | 3 |
|  | 126–150 | 4 |
|  | 101–125 | 5 |
|  | ≤100 | 6 |
| Respiratory rate, breaths/min | ≤30 | 0 |
|  | 31–35 | 1 |
|  | 36–40 | 2 |
|  | 41–45 | 3 |
|  | ≥46 | 4 |

GCS = Glasgow Coma Scale, HACOR = heart rate, acidosis, consciousness, oxygenation, and respiratory rate

^#^The table is referenced from Duan J, Han X, Bai L, et al. Assessment of heart rate, acidosis, consciousness, oxygenation, and respiratory rate to predict noninvasive ventilation failure in hypoxemic patients. Intensive Care Med 2017; 43:192–199.

Supplementary Table 2. Variables left in the elastic net logistic regression for predicting NIV failure in the training cohort from baseline data

| Variable | Regression coefficient β per unit increase | OR (95% CI) | p |
| --- | --- | --- | --- |
| Pneumonia | 0.90 | 2.46 (1.88–3.24) | <0.01 |
| CPE | –1.59 | 0.20 (0.09–0.45) | <0.01 |
| Presence of pulmonary ARDS | 1.11 | 3.05 (2.06–4.51) | <0.01 |
| Presence of immunosuppression | 0.54 | 1.72 (1.12–2.65) | 0.01 |
| Presence of septic shock | 0.96 | 2.61 (1.76–3.87) | <0.01 |
| SOFA score | 0.19 | 1.21 (1.15–1.28) | <0.01 |

NIV = noninvasive ventilation, OR = odds ratio, CI = confidence interval, CPE = cardiogenic pulmonary edema, ARDS = acute respiratory distress syndrome, SOFA = sequential organ failure assessment

Supplementary Table 3. Basic score for predicting NIV failure in the training cohort

| Variable | Regression coefficient β per unit increase | Weight (β/β_reference_) × 0.5 | Assigned points |
| --- | --- | --- | --- |
| Pneumonia | 0.90 | 0.90/0.19 × 0.5 = 2.37 | 2.5 |
| CPE | –1.59 | –1.59/0.19 × 0.5 = –4.18 | –4 |
| Presence of pulmonary ARDS | 1.11 | 1.11/0.19 × 0.5 = 2.932 | 3 |
| Presence of immunosuppression | 0.54 | 0.54/0.19 × 0.5 = 1.42 | 1.5 |
| Presence of septic shock | 0.96 | 0.96/0.19 × 0.5 = 2.53 | 2.5 |
| SOFA score | 0.19 | 0.19/0.19 × 0.5 = 0.5 | 0.5 × SOFA |

NIV = noninvasive ventilation, CPE = cardiogenic pulmonary edema, ARDS = acute respiratory distress syndrome, SOFA = sequential organ failure assessment

The basic formula for NIV failure is 0.5 × SOFA + 2.5 if pneumonia is diagnosed – 4 if CPE is diagnosed + 3 if pulmonary ARDS is present + 1.5 if immunosuppression is present + 2.5 if septic shock is present

Supplementary Table 4. Combining the basic score and the original HACOR score to predict NIV failure in the training cohort.

| Variable | Regression coefficient β per unit increase | OR (95% CI) | p |
| --- | --- | --- | --- |
| Basic score | 0.32 | 1.37 (1.30–1.44) | <0.01 |
| HACOR score after 1–2 h of NIV | 0.39 | 1.48 (1.40–1.56) | <0.01 |

HACOR = heart rate, acidosis, consciousness, oxygenation, and respiratory rate, NIV = noninvasive ventilation, OR = odds ratio, CI = confidence interval

A unit increase in the basic score is associated with a 1.37-fold increase in NIV failure. A unit increase in the HACOR score after 1–2 h of NIV is associated with a 1.48-fold increase in NIV failure. As the risk for NIV failure per increment increase is close between the basic and HACOR scores, we added the two scores together and called it the updated HACOR score. Thus, the updated HACOR score = original HACOR score + basic score.


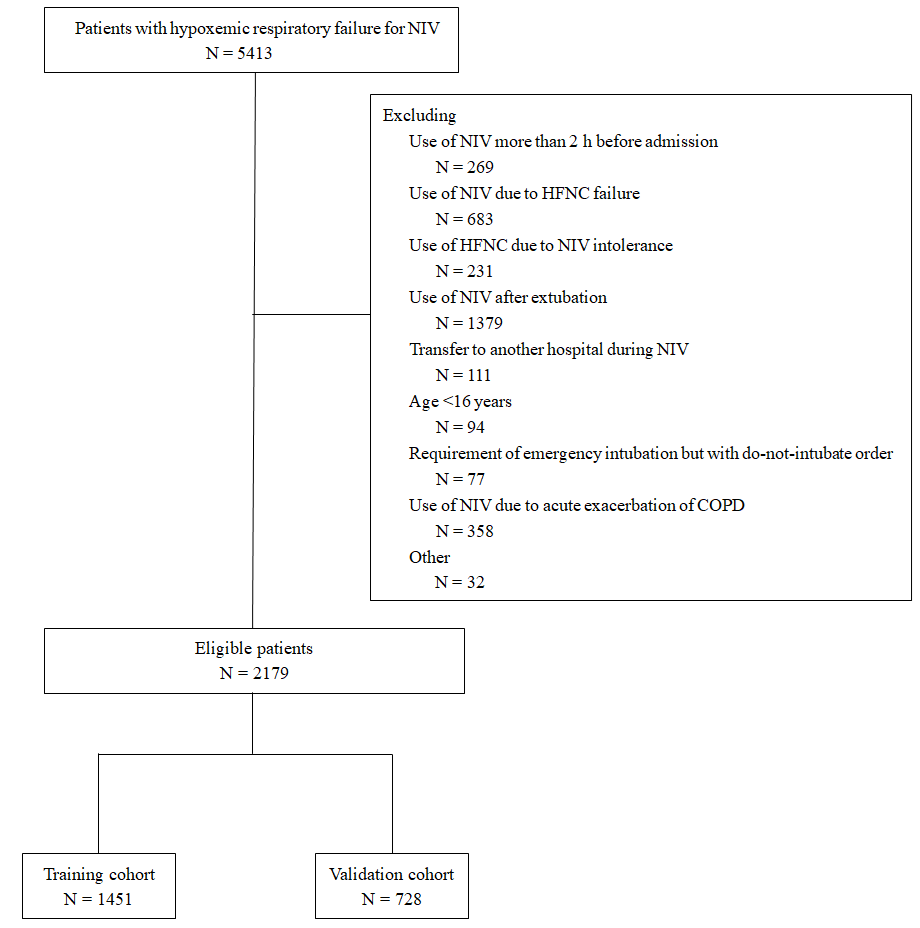


Supplementary Figure 1. Flow of patient screening and enrollment.

NIV = noninvasive ventilation, HFNC = high-flow nasal cannula, COPD = chronic obstructive pulmonary disease.


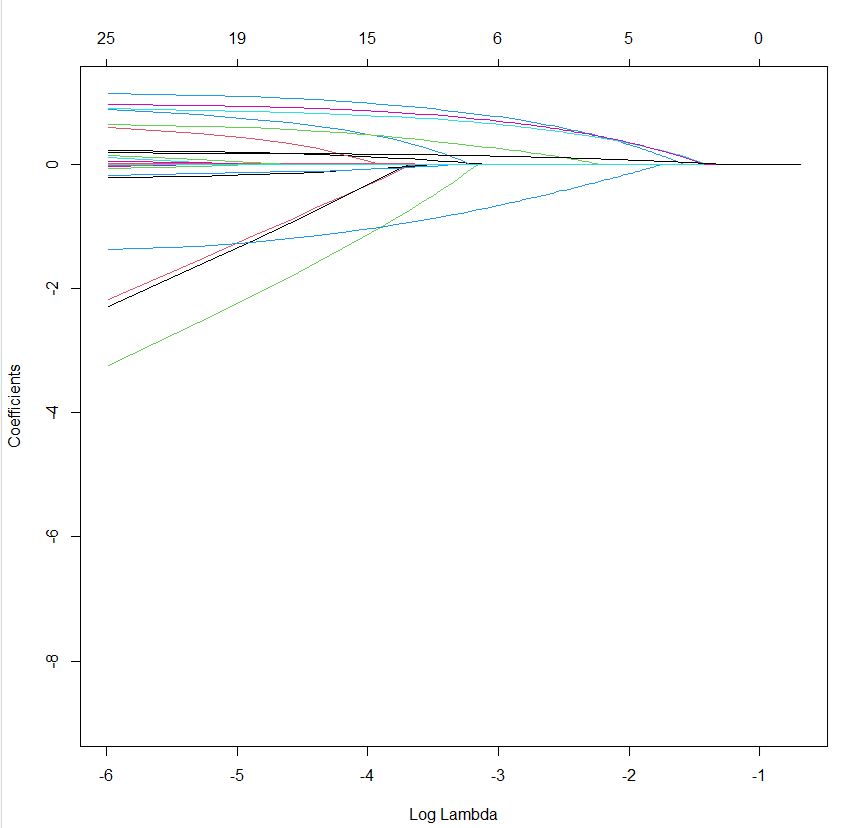


Supplementary Figure 2. Change in variable counts in the elastic net logistic regression.

The number of variables in the elastic net logistic regression was reduced as the penalty coefficient lambda increased. A higher lambda indicates a more severe penalty. The x axis at top indicates the number of predictors for the given log(lambda).


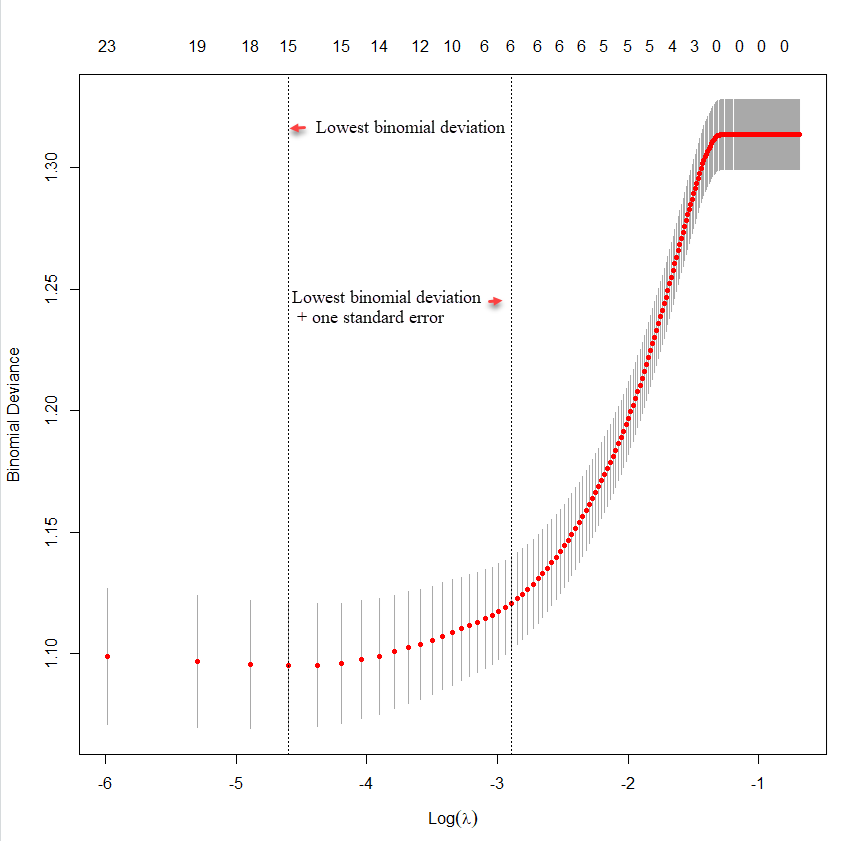


Supplementary Figure 3. The selection of variables in the elastic net logistic regression by 10-fold cross-validation.

The penalty coefficient lambda in the elastic net model was tuned using 10-fold cross-validation and the “lambda.1se” criterion. The x axis at bottom represents the continuous increase in the penalty coefficient lambda, and the x axis at top represents the continuous decrease in the number of variables in the model from left to right. Vertical lines were plotted at the given lambdas selected by 10-fold cross-validation with minimum binomial deviation and minimum binomial deviation plus one standard error, respectively. The optimal lambda was determined at the minimum binomial deviation plus one standard error. At this lambda, six variables with non-zero coefficients were identified.


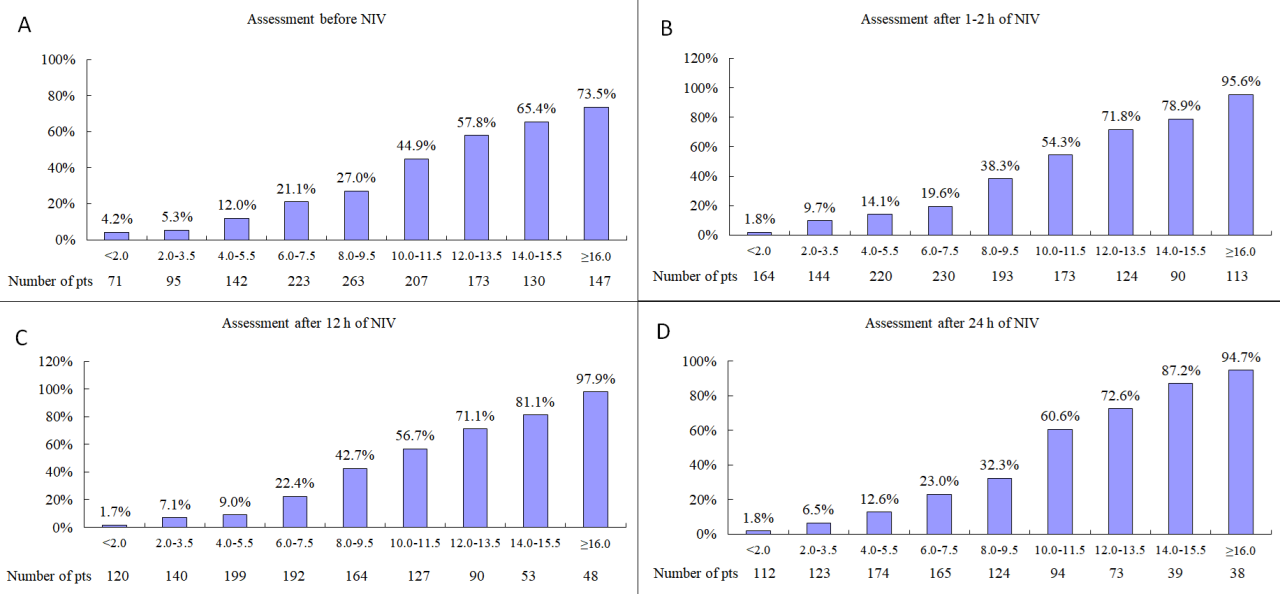


Supplementary Figure 4. Rate of NIV failure in patients in the training cohort with different updated HACOR scores.

NIV = noninvasive ventilation, HACOR = heart rate, acidosis, consciousness, oxygenation, and respiratory rate.


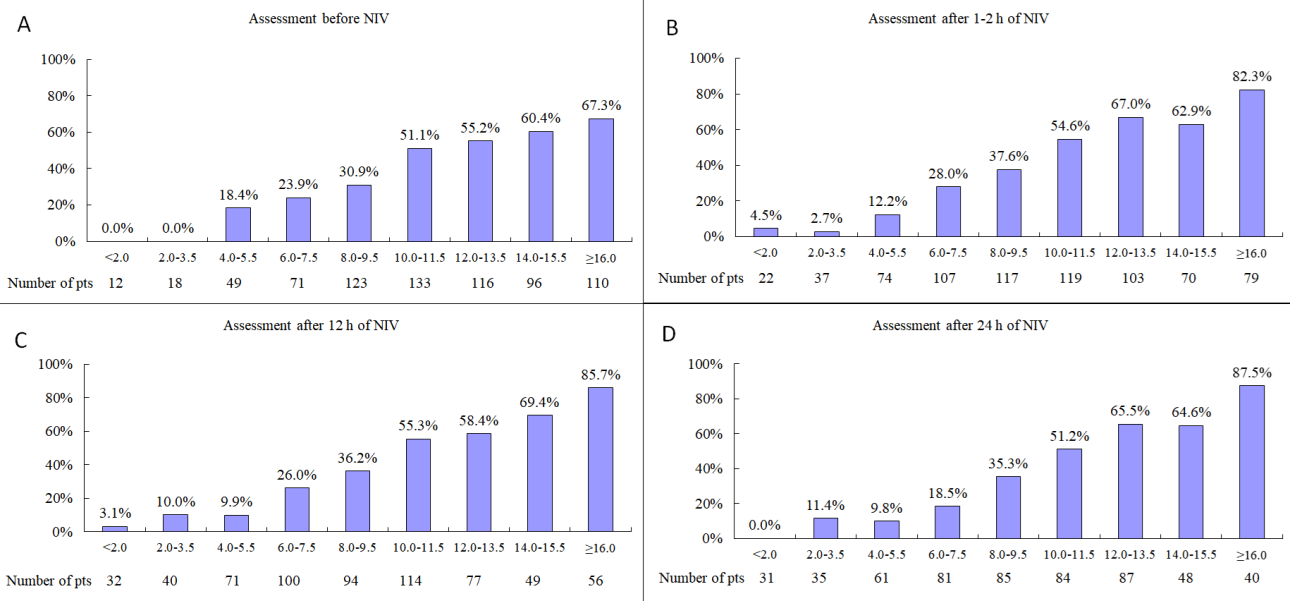


Supplementary Figure 5. Rate of NIV failure in patients in the validation cohort with different updated HACOR scores.

NIV = noninvasive ventilation, HACOR = heart rate, acidosis, consciousness, oxygenation, and respiratory rate.

**Supplementary Method 1. Details of the development of the updated heart rate, acidosis, consciousness, oxygenation, and respiratory rate (HACOR) score in the training cohort**

**Identifying variables associated with the failure of noninvasive ventilation (NIV) from the baseline data**

Before NIV, we collected data on 25 candidate variables at baseline to identify factors associated with NIV failure. The candidate variables were age; sex; sequential organ failure assessment (SOFA) score; and the presence of COVID-19, septic shock, pneumonia, nonpulmonary sepsis, pancreatitis, cardiogenic pulmonary edema (CPE), cardiac problem other than CPE, embolism, trauma, poison, postoperative respiratory failure, asthma, inhalation injury, pulmonary acute respiratory distress syndrome (ARDS), extrapulmonary ARDS, hypertension, diabetes mellitus, chronic kidney disease, chronic liver disease, chronic heart disease, chronic lung disease, and immunosuppression. No collinearity between continuous variables was identified [1].

We selected variables via elastic net regularization, using logistic models and 10-fold cross-validation, selecting the regularization parameter λ when binomial deviation was within one standard error of the minimum [2,3]. The number of variables in the model was reduced as the penalty coefficient lambda increased (Supplementary Figure 2). The lowest binomial deviation was determined with 10-fold cross-validation (Supplementary Figure 3). Then we calculated the value of one standard error. The optimal value was determined with lowest binomial deviation plus one standard error [3]. Ultimately six variables strongly associated with NIV failure before the initiation of NIV were identified. They were the SOFA score, diagnoses of pneumonia and CPE, the presence of pulmonary ARDS, immunosuppression, and septic shock (Supplementary Table 2).

Using these six variables, we developed a scale (basic score) of NIV failure (Supplementary Table 3). As the regression coefficient beta per unit increase was lowest for the SOFA score, we used the coefficient beta (0.19) as the reference. As the other variables were dichotomous, their weights were coefficient beta divided by the reference value. For convenience, the weight for each variable was multiplied by 0.5. Then it was rounded to the nearest value to determine the assigned points. Because 0.5 points is a 1-unit increase in the SOFA score, the points for SOFA score were determined using 0.5 × SOFA.

Therefore, the basic formula for NIV failure is 0.5 × SOFA + 2.5 if pneumonia is diagnosed – 4 if CPE is diagnosed + 3 if pulmonary ARDS is present + 1.5 if immunosuppression is present + 2.5 if septic shock is present.

**Combining the basic score and original HACOR score to determine the updated HACOR score**

The basic score and the original HACOR score before and after 1–2 h of NIV were entered into an elastic net logistic regression. Ultimately the basic score and the original HACOR score after 1–2 h of NIV were left in the model (Supplementary Table 4). The odds ratios of NIV failure were 1.37 and 1.48 per 1-point increase in the basic and original HACOR scores, respectively. The regression coefficient betas per unit increase in the basic and original HACOR scores were 0.32 and 0.39, respectively. As the odds ratio and regression coefficient beta were very close, we added the basic score and the original HACOR score together as the updated HACOR score for convenience.

Ultimately the updated HACOR score is as follows: original HACOR score + 0.5 × SOFA + 2.5 if pneumonia is diagnosed – 4 if CPE is diagnosed + 3 if pulmonary ARDS is present + 1.5 if immunosuppression is present + 2.5 if septic shock is present.

**References**

1. Dormann CF, Elith J, Bacher S, et al. Collinearity: a review of methods to deal with it and a simulation study evaluating their performance. Echography. 2013; 36:27-46.
2. Leisman DE, Harhay MO, Lederer DJ, et al. Development and reporting of prediction models: Guidance for Authors From Editors of Respiratory, Sleep, and Critical Care Journals. Crit Care Med 2020; 48:623-633.
3. Lyu M, Zhou J, Jiao L, et al. Deciphering a TB-related DNA methylation biomarker and constructing a TB diagnostic classifier. Mol Ther Nucleic Acids 2022; 27:37-49.
